# Effects of Conventional and Bokashi Hydroponics on Vegetative Growth, Yield and Quality Attributes of Bell Peppers

**DOI:** 10.3390/plants10071281

**Published:** 2021-06-24

**Authors:** René Clarisse Tong, Charles Stephen Whitehead, Olaniyi Amos Fawole

**Affiliations:** Postharvest Research Laboratory, Department of Botany and Plant Biotechnology, University of Johannesburg, P.O. Box 524, Auckland Park, Johannesburg 2006, South Africa; renet@uj.ac.za (R.C.T.); cswhitehead@uj.ac.za (C.S.W.)

**Keywords:** organic hydroponics, nutrient medium, sustainability, food security, quantum yield, peppers

## Abstract

Due to consumers’ awareness and concern about nutrition and health in different parts of the world, the adoption of organic hydroponics is increasing. This has led to a search for organic nutrient media. One of the viable nutrient sources for organic hydroponics is bokashi compost. The principal objective of this study was to compare the performance of 10% bokashi hydroponics with convention hydroponics for bell pepper production. The different hydroponics influenced vegetative growth parameters largely due to considerable differences in the mineral elements in both hydroponic systems. Stems of conventionally grown plants were significantly (*p* ≤ 0.05) thicker (10.2 mm) compared to those of the bokashi grown plants (7.3 mm). Conventionally grown plants had significantly (*p* ≤ 0.05) higher photosynthetic performance than bokashi grown plants; normalized difference vegetation index (NDVI) (78.80 versus 67.49), soil plant analysis development (SPAD; 73.89 versus 38.43), and quantum yield (QY; 0.64 versus 0.49). Leaf superoxide dismutase (SOD) activity in the leaves of bokashi grown plants (0.32 units/mg protein) was significantly (*p* ≤ 0.05) lower than in the leaves of conventionally grown plants (0.37 units/mg protein). This also corresponded to significantly (*p* ≤ 0.05) higher leaf sap content in the conventionally grown plant than bokashi grown plants. Furthermore, conventional hydroponics yielded three-fold greater pepper fruit per plant compared to bokashi. After 14 days of storage at 7 °C and 95% relative humidity, the firmness of both groups declined, especially for the bokashi grown fruit (27.73 shore unit), which was significantly lower compared to conventionally grown fruit (35.65 shore unit). However, there was an increase in carotenoid content in fruit grown in both hydroponic systems after storage. In conclusion, although bell pepper plant was successfully cultivated in bokashi hydroponics, the plant performance, fruit yield and postharvest quality were lower than conventional hydroponics. We believe that this study and its approach will provide future research with baseline information on optimizing media of bokashi hydroponics to produce bell pepper.

## 1. Introduction

Bell pepper (*Capsicum annuum*, L.) is a non-climacteric fruit belonging to the Solanaceae family and is indigenous to Mexico, Central America and America [1]. It is an economically important crop in most areas of the world. Bell peppers are an important source of antioxidants, such as carotenoids and vitamin C [2]. Although bell peppers are usually grown in the soil, some farmers have opted to switch to soilless cultivation, mainly hydroponics. The main reason for this switch is linked to achieving higher yields, better fruit quality, and sustainable farming due to environmental concerns [3]. In conventional hydroponics, minerals are supplied in the form of a carefully formulated inorganic nutrient solution. However, due to consumers’ awareness and concern about nutrition and health in different parts of the world, the adoption of organic hydroponics is increasing [4].

One of the viable nutrient sources for organic hydroponics is bokashi compost. Bokashi compost is a fermented product consisting of wheat or rice bran, oil cake, manure, molasses and effective microorganisms [5]. Effective microorganisms consist mainly of lactic acid bacteria, photosynthetic bacteria, yeasts, actinomycetes, and fermenting fungi [5]. Previous studies have reported the benefits of applying bokashi compost with concentrations between 10% and 30% as an amendment to soil for the cultivation of various crops [6,7]. For instance, Maass et al. [7] reported that 15% and 30% resulted in higher chlorophyll index and foliar phosphate higher yields. In another study by Pohan et al. [6], 30% of bokashi for the production of tomatoes increased the plant height and stem diameter by 60% and 50%, respectively, and yield by 60%. Some of the advantages of bokashi are that it is odorless, plays a role in pathogen resistance, improves soil quality, and is a sustainable way of using biowaste for compost [5].

Studies on the use of bokashi for plant production have focused on its use as an amendment to soil with no information on the potential use of bokashi in hydroponics. The concept of bokashi hydroponics is based on the method of Kyan et al. [5]. According to the author, in bokashi hydroponics, plants are grown in an inert medium, and nutrients are supplied by bokashi compost mixed with the medium in a fixed ratio and topped up at fixed intervals during the production period [5].

To the best of our knowledge, no study has investigated the potential of a bokashi hydroponic system for the cultivation or growth of bell peppers. Based on our preliminary study, the pH, electrical conductivity, and salt content of 10% bokashi favored survival and growth of bell pepper. Therefore, this study aimed to investigate the potential of a 10% bokashi hydroponic system by comparing its performance with conventional hydroponics based on vegetative growth, yield, and postharvest quality of bell peppers (cv. King Arthur).

## 2. Results and Discussion

### 2.1. Nutrients in Growth Media

Macronutrients in the leachate from the bokashi compost, except potassium and phosphates, were significantly (*p* ≤ 0.05) lower than in the conventional commercial nutrient solution. In addition, the micronutrients, including manganese, copper, iron and zinc, were significantly lower in the bokashi leachate than in conventional hydroponics (Table 1).

Similarly, nitrate, calcium, magnesium and sulphate macronutrients were significantly (*p* ≤ 0.05) lower. In contrast, micronutrients such as sodium, chloride and copper were higher in bokashi hydroponics than in the conventional medium. Although not statistically (*p* > 0.05) significant, this suggests that plants grown in the bokashi medium could be subjected to salt stress [8]. The higher levels of these two minerals could be ascribed to poultry manure in the bokashi compost [9]. It is also evident from the results that ammonium appears to be the major nitrogen source for plants grown in bokashi compost because the nitrate level was significantly lower (Table 1). Again, the high level of ammonium in the bokashi medium could be ascribed to its high levels in poultry manure [10]. According to Jones [10], high ammonium levels can have detrimental effects on plants by depleting carbohydrates in the roots and induced deficiency of cations such as K^+^ over time.

### 2.2. Effect of Bokashi and Conventional Hydroponics on Vegetative Growth Parameters

#### 2.2.1. Plant Height, Stem Diameter and Internodes

Plants grown with conventional hydroponics were significantly (*p* < 0.05) taller (762 mm) compared to the bokashi grown plants (611 mm) (Table 2).

Although not statistically significant (*p* > 0.05), the difference in plant height could be due to the high level of Na+ in the bokashi medium, which could result in salinity stress [11]. The stunted growth could also be due to nutrient deficiencies. For example, Razaq et al. [12] and Silva et al. [13] reported that nitrogen deficiency in soybean plants and mint plants (*Mentha piperita*) resulted in shorter stems and ultimately shorter plants, respectively. There was no significant (*p* ≤ 0.05) difference between the internode lengths in both conventional (2.95 mm) and bokashi grown plants (2.65 mm). However, the stems of conventionally grown plants were significantly (*p* ≤ 0.05) thicker (10.2 mm) compared to those of the bokashi plants (7.3 mm) (Table 2). As explained for plant height, the bokashi grown plants’ thinner stem could also be ascribed to nutrient deficiencies and salinity [13].

#### 2.2.2. Photosynthetic Performance

In comparison, soil plant analysis development (SPAD) and normalized difference vegetation index (NDVI) values were significantly higher (*p* ≤ 0.05) in plants grown conventionally than in those grown in the bokashi hydroponics (Table 2). SPAD is widely used for the rapid, accurate and non-destructive measurement of leaf chlorophyll concentrations, and it is reported to differ from solvent-extracted chlorophyll by just ~6% on average [14]. The low SPAD value in bell pepper plants grown with a bokashi medium could be ascribed to the early chlorophyll breakdown during senescence. This could be attributed to a deficiency of minerals required for chlorophyll synthesis in plants, especially iron, nitrogen and magnesium, which were lower in the bokashi medium (Table 1). For instance, Riga and Anza et al. [15] reported that under Mg-deficiency, pepper plants decrease in photosynthetic productivity, resulting in low growth rate, total dry weight, stem and root mass fractions, total leaf area, specific leaf area, and unit leaf rate. Like SPAD measurement, the NDVI values of plants grown by conventional hydroponics were higher than those of bokashi grown plants (Table 2). Lower NDVI values are an indication of stress in plants [16]. As mentioned earlier, such stress could be attributed to the deficiency of certain minerals and the high sodium level in the bokashi medium. Quantum yield (QY), a measure of the efficiency of photosystem II (PS II) [17], was significantly lower in bokashi (0.49) than in conventionally grown plants (0.62) (Table 2). The lower QY suggests a higher stress level due to deficiency in minerals in the bokashi grown plants [18]

#### 2.2.3. Leaf Superoxide Dismutase (SOD)

According to Ighodaro and Akinloye [19], SOD is the first detoxification enzyme and most powerful antioxidant in the cell. It is an important endogenous antioxidant enzyme that acts as a first-line defense system against reactive oxygen species (ROS). It catalyzes the dismutation of two molecules of superoxide anion (^∗^O_2_) to hydrogen peroxide (H_2_O_2_) and molecular oxygen (O_2_), consequently rendering the potentially harmful superoxide anion less hazardous. The SOD activity in the leaves of bokashi grown plants was significantly (*p* ≤ 0.05) lower than in the leaves of conventionally grown plants (Table 2), suggesting its lower ability to scavenge ROS than in conventionally grown plants. According to Marschner [20], zinc deficiency results in a decrease in CuZnSOD. Thus, it is logical to hypothesize that a severe mineral deficiency resulted in the lower SOD activities in the leaves of bokashi plants, because the levels of both these minerals are deficient in the bokashi medium. The high levels of Na and Cl in the bokashi medium (Table 1), although not significant (*p* ≤ 0.05), could also have impacted the low levels of SOD activity. This is in agreement with Clark et al. [21], who reported that the activity of CuZnSOD decreased when licorice plants were exposed to high NaCl concentration.

#### 2.2.4. Leaf Sap Content

The nitrate and potassium levels in the leaf sap were significantly (*p* ≤ 0.05) higher in the leaves of conventional hydroponic than in bokashi grown plants, whereas the phosphate levels in the leaf sap were significantly (*p* ≤ 0.05) lower in the leaves of conventional hydroponic plants (Table 3).

Comparing nitrate uptake with nitrate content in the leachate, conventionally and bokashi grown plants showed nitrate uptakes of 36% and 70%, respectively.

Although the nitrate percentage uptake relative to the supplied nitrate in conventionally grown plant was lower compared to that in bokashi grown plants, the sap analysis showed that the actual level of NO_3_^-^ in the leaves of the conventional hydroponic plants was significantly higher than in the leaves of the bokashi hydroponic plants (Table 3). This is in line with the low level of nitrates in bokashi hydroponics (Table 1). This is consistent with Cometti et al. [22], who reported that low nitrate levels in the nutrient solution resulted in low levels of nitrates in the leaves of lettuce plants. Calcium directly affects nitrogen assimilation due to its role in activating nitrate enzyme activity [23]. Calcium deficiency could also play a role in the lower nitrate levels in the bokashi plants. This corroborates with Lavon et al. [24] who observed that calcium deficiency in citrus plants resulted in lower nitrate levels in the plants.

The potassium concentration (K^+^) in the leaf sap of plants grown with conventional hydroponics was significantly (*p* ≤ 0.05) higher compared to the leaf sap of the plants grown with bokashi hydroponics (Table 3). This could be due to the high levels of ammonium in the bokashi medium (Table 1). According to Serna et al. [25], the low levels of K^+^, Ca^2+^ and Mg^2+^ in *Populus x canescens* plants were due to the combined effects of high ammonium levels in the soil (as the main source of nitrogen) on the uptake of these cations. The impairment of K^+^ uptake by high ammonium levels in the growth medium was also observed in rice [26].

The phosphate level in the leaf sap of plants grown with bokashi hydroponics was significantly (*p* ≤ 0.05) higher than those obtained from plants grown with conventional hydroponics (Table 3), although there was no significant difference between the phosphate content in both hydroponic media (Table 3). Again, the high phosphate levels in the sap of bokashi plants can be ascribed to the high ammonium levels (although not statistically significant) in the bokashi medium (Table 1) because phosphate levels in leaves are higher when ammonium is the main source of nitrogen [27].

### 2.3. Effects of Bokashi and Conventional Hydroponics on Fruit Yield, Harvest Quality and Postharvest Performance

#### 2.3.1. Yield, Fruit Size and Weight

The goal to produce comparable yields with bokashi hydroponics was not achieved (Table 4).

The number of fruit produced by the conventionally grown hydroponic plants (8.4) was significantly higher (*p* ≤ 0.05) compared to that of the bokashi plants, which produced approximately 3 times fewer fruit (Table 4). In addition, a total yield of 1.02 kg per plant was obtained using conventional hydroponics, whereas bokashi hydroponics yielded 0.76 kg per plant (Table 4). This means the bokashi grown plant had a 0.34-fold lower yield. Similarly, conventional hydroponics produced fruit with a 1.2-fold circumference (Table 4). The lower yields of the bokashi grown plants can be attributed to the low essential nutrients in the bokashi leachate (Table 1). In addition, the low nitrate content could also affect the fruit yield. According to Jones [4], fruit yields are lower when ammonium is the main source of nitrogen, and this was the case with the bokashi leachate (Table 1). According to the standards set by the South African National Department of Agriculture, fresh bell peppers destined for the fresh market must be at least 80 mm in circumference (Department of Agriculture Forestry and Fisheries 2013).

#### 2.3.2. Fruit Respiration Rate

**Figure 1:**
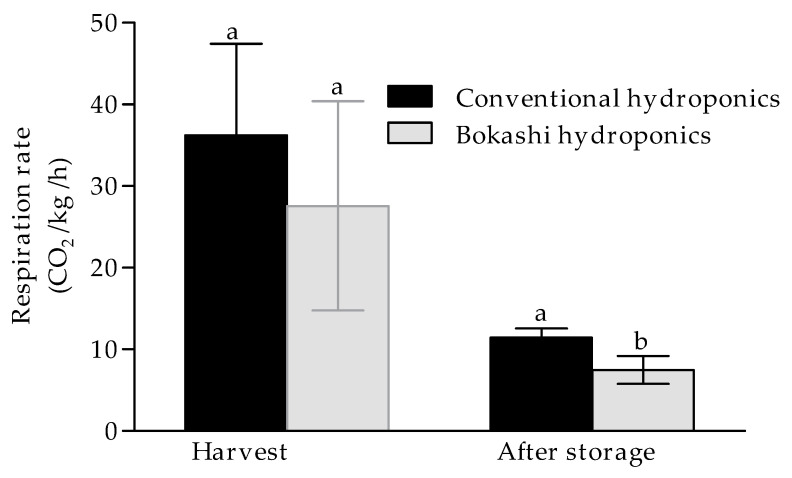
Changes in respiration rate of peppers grown using conventional and bokashi hydroponics, at harvest and after storage. Bell peppers were stored at 7 °C and 95% RH. Data represent the mean and standard deviation of (*n* = 3). Different letters indicate significant differences using *t*-test, two tail at *p* ≤ 0.05.

Respiration is the mitochondrial process where, in the presence of O_2_, sugars are converted to CO_2_ and water with the release of energy [28]. The respiration rate of freshly harvested red ripe fruit produced by conventional hydroponics was on average higher (36.22 mL CO_2_/kg/h) compared to the fruit produced by bokashi hydroponics (27.58 mL CO_2_/kg/h) and not significantly different (Figure 1).

There was a significant decrease in fruit respiration between harvest and after storage for 14 d for ripe bell peppers grown with conventional and bokashi hydroponics. The decrease in respiration rate could be due to the cold temperature storage of the bell pepper compared to the temperature at harvest [29]. However, contrary to the difference at harvest, the respiration rate of bokashi grown fruit was significantly lower (*p* ≤ 0.05) than that of fruit produced with conventional hydroponics after storage, with respiration rates of 7.47 mL CO_2_/kg fresh weight/h and 11.43 mL CO_2_/kg/h, respectively.

#### 2.3.3. Weight Loss

**Figure 2:**
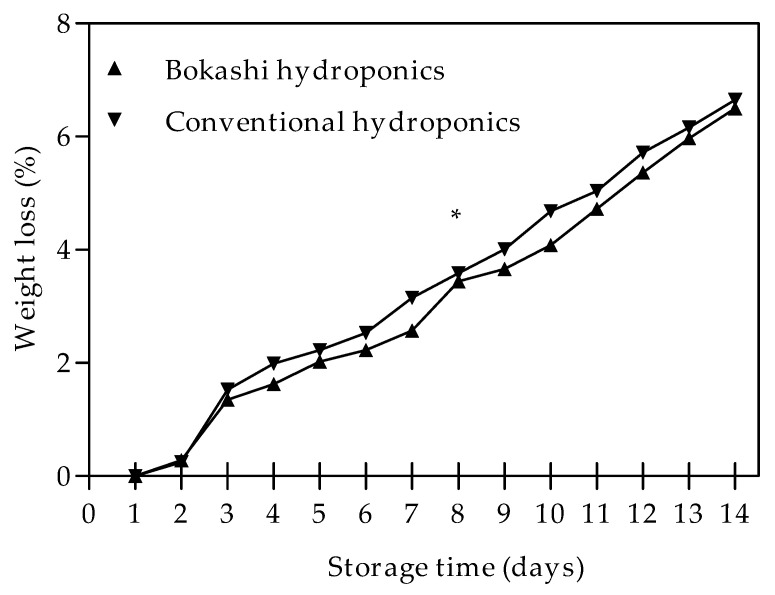
Weight loss of bell peppers grown with conventional and bokashi hydroponics during storage for 14 days at 7 °C and 95% relative humidity. Data represent the mean and standard deviation (*n* = 3). The *t*-test, two tail was used to establish the significant difference between treatments at *p* ≤ 0.05. * = not significant, there are significant differences in daily weight loss between conventional and bokashi grown fruits.

Weight loss increased gradually during the 14 d storage of bell pepper fruit. Weight loss was generally lower in fruit obtained from bokashi hydroponics, albeit not significantly (Figure 2). After 7 days in storage, weight loss values of 3.6% and 3.4% were recorded for the conventional and bokashi grown fruit, respectively. Weight loss further increased to 6.5% in fruit obtained from both bokashi and conventional hydroponics.

#### 2.3.4. Physicochemical Attributes

##### Color and Glossiness

Fruit color is a quality parameter that plays a major role in consumer preference [30]. Therefore, the retention of color after harvest is of prime importance in determining the saleability of the fruit. Except for a* (green-red), there were no significant (*p* ≤ 0.05) differences in the color attributes between ripe fruit grown by conventional and bokashi hydroponics on the day of harvest (Table 5).

After a 14 day storage period, a significant contrast was observed between the two treatments. There was a decrease in L* value for conventional and bokashi grown red-ripe fruit, although the change was not significant for conventionally grown fruit (Table 5). Redness (a*) of bell peppers grown with conventional hydroponics increased (39.10 versus 41.49) after storage, whereas a decrease was observed for bokashi grown fruit. The observed change in redness was significant between both treatments after storage, with conventionally grown fruit exhibiting greater red coloration than bokashi grown fruit (Table 5). At harvest, there were no significant differences in fruit yellowness (b*), color intensity (C*), hue angle (h°) and glossiness between conventional and bokashi grown fruit (Table 5). However, after a 14 d storage, conventionally grown fruit exhibited significantly (*p* ≤ 0.05) higher yellow (increased b*) appearance, intense color (increased C*), increased purity (reduced h°) and glossiness, in comparison with bokashi grown fruit (Table 5). The observed contrast was due to the significant (*p* ≤ 0.05) decline in color attributes (b*, C* and glossiness) and increase in hue angle in bokashi grown fruit between harvest and after a 14 d cold storage (Table 5). The significant decline in bokashi grown peppers’ color parameters, which were indeed visually darker compared with conventionally grown fruit after storage, could indicate signs of oxidative browning. A similar observation was reported by Frans et al. [31], who reported color degradation as a result of oxidative browning in control samples compared with MAP packed red fruit peppers. According to Osuna-Garcia et al. [32], oxidative browning in pepper results from the degradation of carotenoids, which are prone to oxidation because of conjugated double bonds in their structure.

##### Firmness and Moisture Content

Texture is another major distinguishing attribute of fruit quality and plays an important role in determining a fruit’s postharvest life duration. Both firmness and moisture content contribute to a fruit’s texture. There were no significant differences at harvest between firmness and moisture content of fruit grown with conventional and bokashi hydroponics (Table 6).

After 14 days of storage at 7 °C and 95% relative humidity, the firmness of both groups declined, especially for the bokashi grown fruit (27.73 shore unit), which was significantly lower compared to conventionally grown fruit (35.65 shore unit) (Table 6). Compared with initial values at harvest, the observed decline in firmness after cold storage was statistically different in both groups (Table 6). The reduction of firmness could be attributed to polysaccharide degradation and central vacuole disruption in pepper due to biochemical processes [33].

The moisture content declined after storage. The decline in moisture content was a reflection of fruit weight loss after storage. (Figure 2). In comparison, however, there was no significant difference between the conventional hydroponics and the bokashi hydroponics, and the decline observed after cold storage was also not significant for either treatment (Table 6).

##### Total Soluble Solids (TSS)

There was no significant difference in TSS between conventional and bokashi grown fruit at harvest (Table 6). However, after 14 days of storage, there was an increase in TSS (from 9.78 to 10.76 °Brix) for conventionally grown fruit, which resulted in a significantly higher TSS compared with bokashi grown fruit, and a decline in TSS after storage (Table 6). However, the changes in TSS in the individual treatment were not significantly different between harvest and cold storage. Although not statistically significant, the observed TSS behavior in conventionally grown fruit could be due to hydrolytic conversion of complex polysaccharides into simpler sugars and the transformation of pectic substances and juice concentration [34]. Although the decline observed in TSS for bokashi was not significant, the only logical explanation could be a slowdown in metabolic activity and starch breakdown during the storage period [35].

##### Carotenoid Concentration

Carotenoids form part of the antioxidant system of plants and play a vital role in protecting the plant against oxidative stress [36]. Therefore, there is a close relationship between carotenoid content and antioxidant activity [36]. There was no significant difference between conventionally and bokashi grown fruit at harvest and after storage (Table 6). A slight increase in carotenoid concentration was observed for the individual group between harvest and after storage (Table 6). Although the increase was not significant, the higher carotenoid level after storage can be attributed to the ongoing biosynthesis of carotenoids as the fruit ripens [37]. It can be hypothesized that the slow carotenoid biosynthesis in the bokashi fruit could be attributed to stress [38]

## 3. Materials and Methods

### 3.1. Preharvest Conditions

A greenhouse experiment was conducted at the University of Johannesburg, Kingsway Campus, South Africa. The experiment was carried out between February to November under 13 h light and 11 h dark periods at 22–26 °C and 60% relative humidity. Randomized complete block design with two growing media, five replicates and 5 plants in each replication was used. The growing medium for the conventional hydroponics was vermiculite (50%) and perlite 50%, and for bokashi was perlite (40%) + coco coir (40%) + 10% bokashi compost. The bokashi compost consisted of oil cake, wheat bran, molasses, efficient microorganisms, and chicken manure (Table 7).

The vermiculite was substituted with the coconut coir, because it is an organic medium, derived from the husks of coconuts, whereas vermiculite is a silica-based derivative [10]. Bell pepper (*Capsicum annuum cv.* King Arthur) seeds were sown in seed trays. Seedlings were transplanted at the four-leaf stage into 5 L pots filled with the corresponding substrates for conventional hydroponics and bokashi. The coco coir was thoroughly rinsed, and excess liquid was squeezed out before the seedlings were transplanted into the pots containing the bokashi compost. This was done to ensure that there were no excess salt in the media. The conventional plants were supplied with nutrients using a commercial hydroponic nutrient solution (Shiman Hydroponic mix and Omnia Nutriology calcium nitrate, Johannesburg, South Africa) and water in a drip irrigation to the individual bokashi plants and through a pressure pump.

### 3.2. Harvest and Postharvest Conditions

The same randomized complete block design with two growing media, five replicates and 5 plants in each replication was used for postharvest trials.

### 3.3. Assessments of Vegetative Growth Parameters

#### 3.3.1. Plant Height, Stem Diameter and Internodes

Sixty days after transplanting, the plant height was measured from the base to the top using a meter tape. A Vernier caliper was used to measure the stem diameter starting at the bottom of the first internode closest to the base of the plant. Similarly, lengths between different internodes were measured using a Vernier caliper and ruler. Five replicates from each treatment were used in all measurements.

#### 3.3.2. Photosynthetic Assessments

Normalized difference vegetation index (NDVI,) of leaves at different ages from plants grown in conventional and bokashi hydroponics was measured using a portable PlantPen NDVI 300 m. (Photon Systems Inc., Drasov, Czech Republic). Readings were obtained from 5 different conventionally and bokashi grown plants [39].

Quantum yield (QY) of leaves at different ages from plants grown in both conventional and bokashi hydroponics was measured using a FluorPen FP 100 (Photon Systems Inc., Drasov, Czech Republic) portable handheld chlorophyll fluorescence meter. Five different replicates for each treatment were used [40].

The Soil Plant Analysis Development (SPAD) value of leaves at different positions on the stem from plants grown in conventional and bokashi hydroponics was obtained with a Minolta SPAD 502 chlorophyll meter (Konica Minolta Sensing Inc., Tokyo, Japan). The readings were obtained from 5 different conventionally and bokashi grown plants [41].

#### 3.3.3. Leaf Sap Analysis

Three plants were randomly selected from both conventional and bokashi plants. The most recently matured leaf (fourth leaf) from each plant was used to determine the nitrate, potassium and phosphate content. The sap was extracted and prepared, as described by Miller [42]. The nitrate and potassium content of leaf liquid was determined using selective ion meters (Horiba Cardy Selective Ion Meters, Kyoto, Japan), and the phosphate content was determined using a photometer (Hanna C99 Multiparameter, Padova, Italy) [43].

#### 3.3.4. Superoxide Dismutase (SOD)

The SOD activity in leave samples was determined in triplicate spectrophotometrically using a SOD assay kit (Sigma Aldrich, catalogue nr. 19,160 SOD, Johannesburg, South Africa). According to the manufacturer’s instructions, the analysis was conducted and calculated as units/mg protein [30].

### 3.4. Harvest and Postharvest Assessments of Fruit Quality Attributes

Ripe fruit were harvested 106 days after sowing (DAS). Fruit were transferred to the laboratory to analyze quality attributes at harvest before storage at 7 °C and 95% relative humidity for 14 days to assess the postharvest performance.

#### 3.4.1. Weight Loss and Respiration Rate

Weight loss of the peppers was measured by calculating the difference between the initial weight and the final weight and expressed as a percentage (%).

The respiration rate of the fruit was measured in a closed system at 20 °C. The fruit was incubated in an airtight container for 20 min before measuring CO_2_ concentration using an infrared gas analyzer (IRGA, Model No. S 151. Qubit systems Inc., Kingston, Canada). Respiration rate was calculated as mL CO_2_ produced per kg fruit per hour [44].

#### 3.4.2. Physicochemical Attributes

##### Color and Glossiness

External color of the fruit pericarp surface was measured in the CIE L*, a*, b* with a Konica Minolta cr-10 chromameter (Konica Minolta, Tokyo, Japan). Fruit glossiness was measured on opposite sides of the fruit with a gloss meter (Konica Minolta 268, Tokyo, Japan). Chroma (C*) and hue angle (h°) were calculated as shown below (Equations (1) and (2)) according to Pathare et al. [45]:

C* = (a^*2^ + b^*2^)^1/2^ (1)

h° = tan^−1^ (b*/a*) (2)

##### Firmness

The firmness of the peppers was evaluated using a Mextech Shore hardness tester (Model no. SH 6510 A, BSK Technologies, Mumbai, India). A set of five replicates was used for both conventionally and bokashi grown peppers [46].

##### Total Soluble Solids (TSS)

Juice from five fruit pressed with a garlic press was used to determine the TSS (°Brix) using a digital refractometer (Spectrum Technologies, Fort Worth, TX, USA).

##### Moisture Content

A lobe from ripe fruit was weighed and dried in an air-circulating oven at 80 °C until constant weight was achieved in five replicates. The percentage moisture was calculated as follows (Equation (3)) [47]:

Moisture content (%) = [(Initial weight − final weight)/initial weight] × 100 (3)

##### Carotenoid Content

Carotenoids in pepper were qualified by extracting 1 g of fruit pericarp tissue in 10 mL of acetone. Then, the homogenate was diluted with 4 parts acetone and centrifuged for 5 min at 20,000 rpm. The absorbance of the resulting supernatant was then measured spectrophotometrically at 460 nm and results reported in µg per gram dry matter.

## 4. Statistical Analysis

StatsPlus 2007 version (AnalystSoft Inc., Alexandria, VA, USA) was used for statistical analysis. Data are represented as mean and standard deviation (*n* = 5). Significant differences were established using the *t*-test, two tail at *p* ≤ 0.05. GraphPad Prism software 4.03 (GraphPad Software, Inc., San Diego, CA, USA) was used for graphical presentations.

## 5. Conclusions

Although bell pepper plants were successfully cultivated in 10% bokashi hydroponics, the performance of the plant grown in this hydroponic system was not comparable with that using conventional hydroponics. The bokashi hydroponic system resulted in plants with thinner stems, and lower photosynthetic and SOD activity. As a result, the goal to produce comparable yields with bokashi hydroponics was not achieved, and bokashi grown fruit were less marketable after a 14 d storage. Nevertheless, our findings showed that the use of bokashi as an organic hydroponic is promising if optimized. Based on our preliminary study, only 10% bokashi favored the survival and growth of bell pepper plants. This was linked to the favorable pH, electrical conductivity, and salt content of the 10% bokashi medium. It is believed that the performance of bokashi could have been influenced by differences induced by the use of coir rather than vermiculite in this study because vermiculite is not considered organic. Nonetheless, this study and its approach will provide future research with baseline information on optimizing raw materials used in bokashi hydroponic systems to produce bell pepper.

## Figures and Tables

**Table 1:** Mineral nutrient analysis of leachate from 10% bokashi compost and conventional commercial hydroponic systems.

Nutrient Composition (mg/L)	Conventional	Bokashi	*p* Value
Chloride (Cl)	29.00 ± 0.00 ^a^	125.33 ± 142.28 ^a^	0.2687
Sulphate (SO_4_)	213.00 ± 0.00 ^a^	57.67 ± 44.64 ^b^	0.0060
Nitrate (N)	163.00 ± 0.00 ^a^	24 ± 0.21 ^b^	<0.0001
Ortho Phosphate (P)	55.00 ± 0.00 ^a^	32.08 ± 22.32 ^a^	0.1323
Free and Saline Ammonium (NH_4_^+^)	21.00 ± 0.00 ^a^	54.90 ± 51.47 ^a^	0.2793
Sodium (Na)	11.00 ± 0.00 ^a^	45.00 ± 33.97 ^a^	0.1391
Potassium (K)	255.00 ± 0.00 ^a^	178.25 ± 147.34 ^a^	0.3740
Calcium (Ca)	147.00 ± 0.00 ^a^	8.42 ± 1.34 ^b^	<0.0001
Magnesium (Mg)	34.00 ± 0.00 ^a^	7.42 ± 2.51 ^b^	0.0002
Boron (B)	1.11 ± 0.00 ^a^	0.85 ± 0.79 ^a^	0.5558
Copper (Cu)	0.05 ± 0.00 ^a^	0.07 ± 0.04 ^a^	0.6322
Iron (Fe)	1.72 ± 0.00 ^a^	0.84 ± 1.25 ^a^	0.2514
Manganese (Mn)	0.45 ± 0.00 ^a^	0.10 ± 0.07 ^b^	0.0017
Zinc (Zn)	0.51 ± 0.00 ^a^	0.19 ± 0.18 ^b^	0.0363

Data represent the mean and standard deviation (*n* = 3). Different letter(s) in each row indicate significant differences using *t*-test, two tail at *p* ≤ 0.05.

**Table 2:** Effects of conventional and bokashi hydroponics on vegetative growth parameters (plant height, stem diameter, stem internode length), photosynthetic performance (NDVI, QY and SPAD).

Growing Substrate	Plant Height (mm)	Stem Internode (mm)	Stem Diameter (mm)	NDVI	QY	SPAD	SOD in Plant (Units/mg Protein)
Conventional	762.6 ± 65.16 ^a^	2.95 ± 1.05 ^a^	10.18 ± 1.14 ^a^	78.80 ± 2.01 ^a^	0.64 ± 0.03 ^a^	73.89 ± 9.98 ^a^	0.37 ± 0.02 ^a^
Bokashi	611 ± 24.34 ^b^	2.60 ± 0.10 ^a^	7.32 ± 0.81 ^b^	67.49 ± 4.13 ^b^	0.49 ± 0.03 ^b^	38.43 ± 10.35 ^b^	0.32 ± 0.02 ^b^
*p* value	0.0012	0.3053	<0.0001	<0.0001	<0.0001	<0.0001	0.0194

Data represent the mean and standard deviation (*n* = 5). Different letters in each column indicate significant differences using *t*-test, two tail, at *p* ≤ 0.05. NDVI; normalized difference vegetation index, QY; quantum yield, SPAD; soil plant analysis development, SOD; superoxide dismutase.

**Table 3:** Nitrate, phosphate and potassium levels in the sap of the matured leaves of bell pepper plants grown by conventional and bokashi hydroponics.

Nutrient Composition (g/L)	Conventional	Bokashi	*p* Value
Nitrate (N)	59.17 ± 7.64 ^a^	17.25 ± 8.98 ^b^	0.0035
Ortho phosphate (P)	1.10± ±0.10 ^a^	1.73 ± 0.03 ^b^	0.0159
Potassium	37.50 ± 2.50 ^a^	17.50 ± 0.08 ^b^	0.0002

Data represent the mean and standard deviation (*n* = 5). Different letters in each column indicate significant differences using *t*-test two tail at *p* ≤ 0.05.

**Table 4:** Effects of conventional and bokashi hydroponics on fruit yield of bell peppers.

	Yield		
Growing Substrate	Number of Fruit per Plant	Fruit Weight per Plant (kg)	Fruit Circumference (mm)
Conventional	8.4 ± 3.78	1.02 ± 55.60 ^a^	278.00 ± 30.45 ^a^
Bokashi	2.6 ± 0.89	0.76 ± 46.06 ^a^	233.6 ± 26.08 ^b^
*p* value	0.0289	0.1528	0.0203

Data represent the mean and standard deviation (*n* = 5). Different letters in each column indicate significant differences using *t*-test, two tail at *p* ≤ 0.05.

**Table 5:** Color attributes of bell peppers obtained from conventional and bokashi hydroponics at harvest and after storage for 14 days at 7 °C and 95% relative humidity.

Growing Substrate	L*	a*	b*	C*	h°	Gloss (Gloss Units)
Harvest						
Conventional	31.5 ± 1.30 ^a^	39.10 ± 2.23 ^a^	13.82 ± 2.04 ^a^	41.47 ± 1.43 ^a^	19.47 ± 2.75 ^a^	1.04 ± 0.03 ^a^
Bokashi	32.60 ± 0.89 ^a^	32.4 ± 3.34 ^b^	12.60 ± 6.70 ^a^	41.06 ± 1.35 ^a^	17.87 ± 1.13 ^a^	0.9 ± 0.46 ^a^
After storage						
Conventional	30.82 ± 0.94 ^a^	41.59 ± 1.89 ^a^	14.50 ± 1.30 ^a^	44.04 ± 1.52 ^a^	19.22 ± 1.12 ^a^	0.43 ± 0.20 ^a^
Bokashi	30.14 ± 0.94 ^b^	30.32 ± 1,49 ^b^	12.25 ± 1.05 ^b^	32.70 ± 1.11 ^b^	22.00 ± 1.38 ^b^	0.34 ± 0.17 ^b^
Harvest vs. after storage						
conventional	ns	ns	ns	ns	ns	ns
Bokashi	*	*	*	*	*	*

Data represent the mean and standard deviation (*n* = 5). Different letters in each column indicate significant differences between treatments either at harvest or after storage using *t*-test two tail at *p* ≤ 0.05. * = significant, there are significant differences between harvest and after 14 d storage. ns = non-significant, there are no significant differences between harvest and after 14 d storage.

**Table 6:** The effects of conventional and bokashi hydroponics on respiration rate, firmness, moisture content, total soluble solids (TSS) and carotenoids in bell peppers at harvest and after storage for 14 days at 7 °C and 95% RH.

Period Hydroponics	Firmness (Shore Units)	Moisture Content (%)	TSS (°Brix)	Carotenoid Content (µg/g)
Harvest				
conventional	52.3 ± 2.46 ^a^	88.63 ± 0.44 ^a^	9.78 ± 1.39 ^a^	1.22 ± 0.40 ^a^
bokashi	46.58 ± 7.55 ^a^	89.62 ± 1.24 ^a^	8.48 ± 0.54 ^a^	1.20 ± 0.23 ^a^
After storage				
conventional	35.65 ± 8.61 ^a^	78.90 ± 1.2 ^a^	10.76 ± 0.37 ^a^	1.55 ± 0.24 ^a^
bokashi	27.73 ± 9.21 ^b^	78.95 ± 1.82 ^a^	8.30 ± 0.67 ^b^	1.24 ± 0.13 ^a^
Harvest vs. after storage				
conventional	*	ns	ns	ns
bokashi	*	ns	ns	ns

Data represent the mean and standard deviation (*n* = 5). Different letters in each column indicate significant differences between treatments either at harvest or after storage using *t*-test two tail at *p* ≤ 0.05. * = significant, there are significant differences between harvest and after 14 d storage. ns = non-significant, there are no significant differences between harvest and after 14 d storage.

**Table 7:** Percentage composition of bokashi compost.

Bokashi Compost Ingredients	%
Wheat bran	60.5
Oil cake	15.1
Chicken manure	15.1
Water	9.1
Molasses	0.1
Efficient microorganisms	0.1
